# Supplementary material for: Impact of COVID-19 pandemic on emergency department visits and infant health: a scoping review protocol
Source: BMJ Open. 2022 Jul 29;12(7):e061778. doi: 10.1136/bmjopen-2022-061778 (PMC9344596; doi:10.1136/bmjopen-2022-061778)
Supplement: Supplementary data [file bmjopen-2022-061778supp001.pdf]

Appendix 1: Search Strategies

1.1 Search Strategy COVID-19 Emergency Department Visits

Embase Classic+Embase <1947 to 2021 June 22>
Ovid MEDLINE(R) ALL <1946 to June 22, 2021>

1 COVID-19/
2 (exp coronavirus/ or coronavirus*.mp. or corona virus*.mp.) and (wuhan or beijing or shanghai or hubei).mp.
3 ((coronavirus* or corona virus* or coronavirus* or coronaviridae or coronaviridae or betacoronavirus*) adj3 ("19" or "2019")).tw.
4 covid.tw,kw.
5 covid19.tw,kw. or covid 19.kw.
6 sars cov 2.tw,kw.
7 (ncov or n cov).tw,kw.
8 (novel coronavirus* or novel corona virus*).tw,kw.
9 (CoV 2 or CoV2 or sarscov2 or 2019nCoV or novel CoV or wuhan virus*).tw,kw.
10 (Coronavirus Infections/ or Severe Acute Respiratory Syndrome/) and (Pandemics/ or pandemic*.tw,kf.)
11 or/1-
12 exp Emergency Service, Hospital/
13 emergency department*.tw,kf.
14 (emergency room* or emergency visit* or ed visit*).tw,kf. or emergenc*.ti.
15 or/12-14
16 11 and 15
17 infant/ or infant, newborn/
18 (infant* or baby or babies or neonat* or newborn*).tw,kf.
19 17 or 18
20 16 and 19
21 20 use medall Medline
22 coronavirus disease 2019/
23 (Coronavirinae/ or coronavirus*.mp. or corona virus*.mp.) and (wuhan or beijing or shanghai or hubei).mp.
24 ((coronavirus* or corona virus* or coronavirus* or coronaviridae or coronaviridae or betacoronavirus*) adj3 ("19" or "2019")).tw.
25 (covid or covid19).tw.
26 sars cov 2.tw.
27 (ncov or n cov).tw.
28 (novel coronavirus* or novel corona virus*).tw.
29 (CoV 2 or CoV2 or sarscov2 or 2019nCoV or novel CoV or wuhan virus).tw.
30 (coronavirus infection/ or severe acute respiratory syndrome/) and (pandemic/ or pandemic*.tw.)
31 limit 30 to yr="2019 -Current"
32 or/22-29,31
33 exp infant/

34 (infant* or baby or babies or neonat* or newborn*).tw.
35 33 or 34
36 32 and 35
37 emergency ward/
38 emergency department*.tw.
39 (emergency room* or emergency visit* or ed visit*).tw. or emergenc*.ti.
40 emergency health service/
41 or/37-40
42 36 and 41
43 42 use emczd Embase
44 21 or 43
45 remove duplicates from 44
46 45 use medall
47 45 use emczd

Web of Science – June 23, 2021
7
#6 AND #5
Indexes=SCI-EXPANDED, CPCI-S, ESCI Timespan=1900-2021

6
TOPIC:
(infant* or baby or babies or neonat* or newborn*)
Indexes=SCI-EXPANDED, CPCI-S, ESCI Timespan=1900-2021

5
#4 AND #3
Indexes=SCI-EXPANDED, CPCI-S, ESCI Timespan=1900-2021

# 4
TS=(emergency room* or emergency visit* or ed visit* or emergency department*) OR TI=(emergenc*)
OR TS=(emergency service*)
Indexes=SCI-EXPANDED, CPCI-S, ESCI Timespan=1900-2021

3
#2 OR #1
Indexes=SCI-EXPANDED, CPCI-S, ESCI Timespan=1900-2021

2
TOPIC: (sars cov 2 OR sars cov2) OR TOPIC: (ncov or n cov)
OR TOPIC: (novel coronavirus or novel corona virus)
Indexes=SCI-EXPANDED, CPCI-S, ESCI Timespan=1900-2021

1
TOPIC: (covid OR covid19 or covid2019)

Indexes=SCI-EXPANDED, CPCI-S, ESCI Timespan=1900-2021

Cinahl - June 23, 2021

Wednesday, June 23, 2021 6:25:31 PM

#	Query
S1	(MH "COVID-19")
S2	(MH "COVID-19 Pandemic")
S3	(MH "SARS-CoV-2")
S4	TI covid* OR AB covid* OR TI sars cov 2 OR AB sars cov 2 OR TI novel coronavirus OR AB novel coronavirus OR TI wuhan virus OR AB wuhan virus OR TI 2019nCoV OR AB 2019nCoV
S5	TI (CoV 2 or CoV2 or sarscov2) OR AB (CoV 2 or CoV2 or sarscov2)
S6	S1 OR S2 OR S3 OR S4 OR S5
S7	(MH "Emergency Service+")
S8	emergency department*
S9	(emergency room* or emergency visit* or ed visit*)
S10	TI emergenc*
S11	(MH "Emergency Medical Services")
S12	(S7 OR S8 OR S9 OR S10 OR S11)
S13	(S6 AND S12)
S14	(MH "Infant+")
S15	TI ((infant* or baby or babies or neonat* or newborn*)) OR AB ((infant* or baby or babies or neonat* or newborn*))
S16	S14 OR S15
S17	(S13 AND S16)

1.2 Search Strategy COVID-19 Neonatal Mortality

Embase Classic+Embase <1947 to 2021 June 22>
Ovid MEDLINE(R) ALL <1946 to June 22, 2021>

1 COVID-19/
2 (exp coronavirus/ or coronavirus*.mp. or corona virus*.mp.) and (wuhan or beijing or shanghai or hubei).mp.
3 ((coronavirus* or corona virus* or coronavirus* or coronaviridae or coronaviridae or betacoronavirus*) adj3 ("19" or "2019")).tw.
4 covid.tw,kw.
5 covid19.tw,kw. or covid 19.kw.
6 sars cov 2.tw,kw.
7 (ncov or n cov).tw,kw.
8 (novel coronavirus* or novel corona virus*).tw,kw.
9 (CoV 2 or CoV2 or sarscov2 or 2019nCoV or novel CoV or wuhan virus*).tw,kw.
10 (Coronavirus Infections/ or Severe Acute Respiratory Syndrome/) and (Pandemics/ or pandemic*.tw,kf.)
11 or/1
12 exp Infant Mortality/
13 exp infant death/ or perinatal death/
14 ((infant* or baby or babies or neonat* or newborn* or perinat*) adj3 (death* or mortalit*)).tw,kf.
15 12 or 13 or 14
16 11 and 15
17 16 use medall Medline
18 coronavirus disease 2019/
19 (Coronavirinae/ or coronavirus*.mp. or corona virus*.mp.) and (wuhan or beijing or shanghai or hubei).mp.
20 ((coronavirus* or corona virus* or coronavirus* or coronaviridae or coronaviridae or betacoronavirus*) adj3 ("19" or "2019")).tw.
21 (covid or covid19).tw.
22 sars cov 2.tw.
23 (ncov or n cov).tw.
24 (novel coronavirus* or novel corona virus*).tw.
25 (CoV 2 or CoV2 or sarscov2 or 2019nCoV or novel CoV or wuhan virus).tw.
26 (coronavirus infection/ or severe acute respiratory syndrome/) and (pandemic/ or pandemic*.tw.)
27 limit 26 to yr="2019 -Current"
28 or/18-25,27
29 infant mortality/
30 newborn mortality/
31 perinatal mortality/
32 perinatal death/
33 ((infant* or baby or babies or neonat* or newborn*) adj2 (death* or mortalit*)).tw.

34 or/29-33
35 28 and 34
36 35 use emczd Embase
37 17 or 36
38 remove duplicates from 37
39 38 use medall
40 38 use emczd

Web of Science – June 23, 2021

5 #4 AND #3
Indexes=SCI-EXPANDED, CPCI-S, ESCI Timespan=1900-2021

4 **TOPIC:** ((infant* or baby or babies or neonat* or newborn*) NEAR/5 (death* or mortalit*))
Indexes=SCI-EXPANDED, CPCI-S, ESCI Timespan=1900-2021

3 #2 OR #1
Indexes=SCI-EXPANDED, CPCI-S, ESCI Timespan=1900-2021

2 **TOPIC:** (sars cov 2 OR sars cov2) *OR* **TOPIC:** (ncov or n cov) *OR* **TOPIC:** (novel coronavirus or novel corona virus)
Indexes=SCI-EXPANDED, CPCI-S, ESCI Timespan=1900-2021

1 **TOPIC:** (covid OR covid19 or covid2019)
Indexes=SCI-EXPANDED, CPCI-S, ESCI Timespan=1900-2021

Cinahl – June 23, 2021

**#**	**Query**
S1	(MH "COVID-19")
S2	(MH "COVID-19 Pandemic")
S3	(MH "SARS-CoV-2")
S4	TI covid* OR AB covid* OR TI sars cov 2 OR AB sars cov 2 OR TI novel coronavirus OR AB novel coronavirus OR TI wuhan virus OR AB wuhan virus OR TI 2019nCoV OR AB 2019nCoV
S5	TI (CoV 2 or CoV2 or sarscov2) OR AB (CoV 2 or CoV2 or sarscov2)
S6	S1 OR S2 OR S3 OR S4 OR S5
S7	(MH "Infant Mortality")

S8	(MH "Infant Death")
S9	(MH "Perinatal Death")
S10	TI (((infant* or baby or babies or neonat* or newborn* or perinat*) N3 (death* or mortalit*))) OR AB (((infant* or baby or babies or neonat* or newborn* or perinat*) N3 (death* or mortalit*)))
S11	(S7 OR S8 OR S9 OR S10)
S12	S6 AND S11

Appendix 2: Data Charting Form

Data charting framework

Main category	Subcategory	Description
**Titles**		
**Authors**		
**Year of publication**		
**Study Journal**		
**Study Citation Details**		
**Study objective(s)**		Describe the stated objectives of the studies included in the review
**Study Design**		Specify the types of studies included in the review
**Study Setting**		Specify the geographical areas covered by the studies included in the review<br>Specify the level of medical facility in each study included in the review
**Study Population/Sample size**		Describe the study population and specify the number of participants included in each study
**Eligibility Criteria**		Specify the inclusion and exclusion criteria of each study included in the review
**Study period**		Specify the time period of data collection of each studies included in the review<br>Specify the stage/wave of the pandemic during which each included study was conducted
**Reported primary outcomes (neonates <28 days and infants <1 year-old)**	Frequency of ED visits	Specify the frequency of ED visits before and during the COVID-19 pandemic
	Main Reason(s) for ED visits	Describe the main reasons for ED visits before and during the COVID-19 pandemic
**Reported secondary outcomes (neonates <28 days and infants <1 year-old)**	Change in mortality	Specify if there was an increase or decrease of neonatal/infant mortality during the COVID-19 pandemic compared to pre-pandemic
	Causes of mortality	Describe the main causes of mortality before and during the COVID-19 pandemic
	ED visit length	Specify the ED visit length (in hours) during the COVID-19 pandemic compared to pre-pandemic
	NICU/PICU/HDU admission	Specify the NICU/PICU/HDU admission rate before and during the COVID-19 pandemic
	Reasons for NICU/PICU/HDU admission	Describe the main reasons for NICU/PICU/HDU admission before and during the COVID-19 pandemic

	NICU/PICU/HDU length of stay	Specify the NICU/PICU/HDU length of stay (in days) during the COVID-19 pandemic compared to pre-pandemic
	Infant hospitalization	Specify the infant hospitalization rate before and during the COVID-19 pandemic
	Reasons for infant hospitalization	Describe the main reasons of infant hospitalization before and during the COVID-19 pandemic
