# Supplementary material for: Impact of COVID-19 pandemic on emergency department visits and infant health: a scoping review protocol
Source: BMJ Open. 2022 Jul 29;12(7):e061778. doi: 10.1136/bmjopen-2022-061778 (PMC9344596; doi:10.1136/bmjopen-2022-061778)
Supplement: Author's manuscript [file bmjopen-2022-061778.draft_revisions.pdf]

BMJ Open

BMJ Open is committed to open peer review. As part of this commitment we make the peer review history of every article we publish publicly available.

When an article is published we post the peer reviewers' comments and the authors' responses online. We also post the versions of the paper that were used during peer review. These are the versions that the peer review comments apply to.

The versions of the paper that follow are the versions that were submitted during the peer review process. They are not the versions of record or the final published versions. They should not be cited or distributed as the published version of this manuscript.

BMJ Open is an open access journal and the full, final, typeset and author-corrected version of record of the manuscript is available on our site with no access controls, subscription charges or pay-per-view fees (http://bmjopen.bmj.com).

If you have any questions on BMJ Open's open peer review process please email info.bmjopen@bmj.com

Impact of COVID-19 pandemic on emergency department visits and infant health: a scoping review protocol

Journal:	*BMJ Open*
Manuscript ID	bmjopen-2022-061778
Article Type:	Protocol
Date Submitted by the Author:	04-Feb-2022
Complete List of Authors:	Osborne, Brenden; Children's Hospital of Eastern Ontario Research Institute; Ottawa Hospital Research Institute<br>Moorjani-Houle, Mélika; Ottawa Hospital Research Institute; University of Ottawa<br>Fakhraei, Romina; Ottawa Hospital Research Institute; University of Ottawa<br>Walker, Mark; Ottawa Hospital Research Institute; University of Ottawa, Department of Obstetrics and Gynecology<br>Wen, Shi Wu; Ottawa Hospital Research Institute; University of Ottawa<br>Guo, Yanfang; Children's Hospital of Eastern Ontario, BORN Ontario; University of Ottawa, School of Epidemiology and Public Health
Keywords:	COVID-19, Paediatric A&E and ambulatory care < PAEDIATRICS, EPIDEMIOLOGY, Community child health < PAEDIATRICS

SCHOLARONE™
Manuscripts

Impact of COVID-19 pandemic on emergency department visits and infant health: a scoping review protocol

Authors:
Osborne, Brenden[1,2], Moorjani-Houle, Mélika[2,3], Fakhraei, Romina[2,4], Walker, Mark[2,4,5], Wen, Shi-Wu[2,4,5], Guo, Yanfang,[1,2,4,6*]

Affiliations:
1. Children's Hospital of Eastern Ontario Research Institute, Ottawa, Ontario, Canada;
2. OMNI Research Group, Clinical Epidemiology Program, Ottawa Hospital Research Institute, Ottawa, Ontario, Canada;
3. Faculty of Medicine, University of Ottawa, Ottawa, Canada;
4. School of Epidemiology and Public Health, University of Ottawa, Ottawa, Ontario, Canada;
5. Department of Obstetrics and Gynecology, University of Ottawa, Ottawa, Ontario, Canada;
6. Better Outcomes Registry & Network Ontario, Ottawa, Ontario, Canada

E-mail Addresses: bosborne@toh.ca (B. Osborne), mmoorjani@ohri.ca (M. M-Houle), rfakhraei@ohri.ca (R. Fakhraei), mwalker@toh.ca (M. Walker), swwen@ohri.ca (SW. Wen), yguo@bornontario.ca (Y. Guo)

***Corresponding Author:**

Dr. Yanfang Guo
Better Outcomes Registry & Network Ontario
CHEO Research Institute – Centre for Practice Changing Research Building
401 Smyth Road, Ottawa, ON K1H 8L6
T: 613-737-8899 (x 73840)
Email: yguo@bornontario.ca

For peer review only

For peer review only - http://bmjopen.bmj.com/site/about/guidelines.xhtml

ABSTRACT

Introduction

The novel SARS-CoV-2 pandemic has provided a set of unique challenges for pediatric patients requiring emergency care across the globe. Reduction in pediatric emergency department (ED) usage during the COVID-19 pandemic has been widely reported, but no studies to date have consolidated and described what ramifications these reductions may have on neonatal and infant health. This scoping review aims to characterize the impact of the COVID-19 pandemic on infant emergency department visits and neonatal and infant health.

Methods and analysis

A comprehensive literature search will be conducted from March 2020 to June 2021 using the following databases: Embase (OVID), Web of Science (Clarivate Analytics), Medline (Ovid) and CINAHL (EBSCOhost). This scoping review will use a five-step framework to guide the selection, extraction and analysis of data from eligible studies, with an additional sixth step for clinical consultation. Studies in English reporting the effect of the COVID-19 pandemic on infant ED visits, as well as neonatal and infant health will be included for screening. Key findings will be reported according to the Preferred Reporting Items for Systematic Reviews and Meta-Analysis Extension for Scoping Reviews.

Ethics and dissemination

Research ethics board approval will not be required due to the nature of study design. The results of this scoping review will be disseminated through publication in a peer-reviewed journal and presentation at academic conferences.

Strengths and limitations of this study

- This scoping review will address the literature gap on the impact of the COVID-19 pandemic on infant emergency department visits and neonatal and infant health.
- Primary studies included in this review will compare data prior to and during the COVID-19 pandemic in order to accurately assess trend differences.
- A clinical expert will be consulted to inform the applicability of study results.
- Due to the novelty of the COVID-19 pandemic, primary studies reporting data on the subject matter are limited.

For peer review only - http://bmjopen.bmj.com/site/about/guidelines.xhtml

INTRODUCTION

In March 2020 the World Health Organization declared the novel Coronavirus (COVID-19) outbreak a global pandemic, which continues to affect millions globally [1]. As individuals restrict their movements, undergo mandatory social distancing, and work from home, hospitals around the globe are observing a reduced patient load in their emergency departments (ED) [2]. The Centre for Disease Control and Prevention reports a 42% decrease in ED visits in the United States based on weekly means, dropping from 2.1 million per week pre-pandemic to 1.2 million during the pandemic, with the steepest decrease reported in the pediatric patient population [3].

Several studies report a steep decrease in pediatric ED visits since the COVID-19 pandemic began [4–7]. A Canadian study of eleven pediatric centers reported a decrease in ED visits by 58% during the pandemic, compared to estimated rates [8]. The literature highlights that reduced ED usage may be a result of increased lockdown measures, social distancing, and fear of contracting SARS-CoV-2 [5]. Data from Korea highlights the association between increasing government restrictions and a reduction in the number of monthly ED visits [9].

Previous work detailing the effect of the H1N1 pandemic on infant health, morbidity, and mortality may offer insight regarding potential effects of SARS-CoV-2 infection in this population. Studies report delays in presentation to the ED during the H1N1 pandemic (2.8 ± 2.3 days) as well an increase in pediatric intensive care unit (PICU) admissions during this time compared to years prior (0.3% vs 0.1%; 95% CI 0.05%, 0.4%) [10,11]. Children younger than two years of age with a confirmed H1N1 diagnosis were reported to have an increased risk of hospitalization during this period (RR 3.3; 95% CI 1.80, 6.05) [10]. One population-based study in England reported the highest case-fatality rate in their infant (<1year) population, with a fatality rate of 151 per 100,000 cases of H1N1[12].

To date, most studies have focused on collecting or retrospectively analyzing primary data to quantify the reduction in pediatric ED usage during the COVID-19 pandemic. The literature is lacking consensus on the implications of these reductions on infant health outside the context of a confirmed COVID-19 diagnosis, in the general infant population. Given what is known about previous pandemics precipitating adverse effects on infant health [10–12], further knowledge synthesis is required to address the novel situation created by the COVID-19 pandemic. Our objective is to conduct a scoping review to characterize the effect of, and to better understand the impact of the COVID-19 pandemic on infant emergency department visits and secondarily, neonatal and infant health.

METHODS AND ANALYSIS

This scoping review will be conducted according to the methodological framework developed by Arksey and O'Malley and refined by Levac *et al* and the Joanna Briggs Institute (JBI) [13–15]. The steps outline by these frameworks are: (1) identification of the research question, (2) identification of relevant studies, (3) selection of studies, (4) charting the data, and (5) collating, summarizing, and reporting results. As recommended by Arksey and O'Malley [13], we included an additional consultation step with a clinical expert, to find additional sources of information and inform the clinical relevance and value of this review.

For peer review only - http://bmjopen.bmj.com/site/about/guidelines.xhtml

Step 1: Identifying the research question

To help identify the main concepts of the primary review question, the Population/Concepts/Context (PCC) framework is suggested by the JBI [15]. The specific PCC framework for this study is presented in **Table 1**. The primary research question for this review is: what is the current evidence reporting the effect of the COVID-19 pandemic on infant emergency department visits, neonatal, and infant health?

Table 1 Population-Concept-Context

**Population**	All peer-reviewed journal articles including neonates (<28 days) and infants (<1 year) will be included.
**Concepts**	Literature reporting on the frequency/rate and main reasons for neonate/infant ED visits during and before the COVID-19 pandemic will be reviewed. Literature reporting on infant outcomes; infant mortality including neonatal death (< 28 days) and infant death (< 1 year of age), main reasons for infant mortality, infant and neonate hospitalization, main reasons for hospital admission, pediatric or neonatal intensive care unit admission and length of ED visit and/or hospital stay will also be reviewed.
**Context**	The context will be hospital emergency departments. The time-frame is before and during the COVID-19 pandemic (March 2020 and onwards). There will be no restrictions on geographical location.

ED, emergency department.

Step 2: Identifying relevant studies

Our search strategy and database choice will be developed in conjunction with, and refined by a trained medical librarian. Four online databases will be searched, including: Embase (OVID), Web of Science (Clarivate Analytics), Medline (Ovid) and CINAHL (EBSCOhost). Key search terms were developed to capture literature related to the effect of the COVID-19 pandemic on infant ED visits, as well as neonatal and infant health. Truncation and Boolean were used to narrow, widen and combine search parameters as necessary. The finalized search strategies are available in online supplementary appendix 1. The literature search will be carried out in February 2022 and will be updated before the review is submitted for publication.

We agreed to the following eligibility criteria for the initial search:

- Type of publication: journal articles.
- Time frame: during the COVID-19 pandemic (March 2020 onwards).
- Study population: neonates (<28 days of age) and infants (< 1 year of age) presenting to the ED for medical attention.
- Study designs: Analytic epidemiological observational study designs (i.e. cohort studies, case-control studies or cross-sectional design studies), analytical ecological studies (i.e. time series studies), and systematic reviews with or without meta-analyses.

For peer review only - http://bmjopen.bmj.com/site/about/guidelines.xhtml

We will exclude: case reports and series, editorials, commentaries, letters to the editor, abstracts, conference proceedings, book chapters, narrative reviews, non-English studies and any studies that do not compare outcome data collected during the pandemic with a time period prior to the pandemic.

Step 3: Study selection

Records from electronic database searches will be imported into Microsoft Excel to eliminate duplicates. Two reviewers (BO, MMH) will independently screen titles and abstracts to determine study eligibility based on predefined inclusion and exclusion criteria. A second screen of the article in full-text will be performed by two independent reviewers (BO, MMH) to ensure that studies fully meet inclusion criteria and report relevant data. Any discrepancies will be resolved by consensus or through a third reviewer (RF). The results of the screening process will be displayed by a Preferred Reporting Items for Systematic Reviews and Meta-Analyses (PRISMA) flow diagram [16].

Step 4: Charting the data

Data will be extracted into standardized forms in Excel by two reviewers (BO, MMH) and checked for accuracy and completeness by a third reviewer (RF). Whenever necessary, the authors of the original study will be contacted for additional information and clarification of the data. Reviewers will resolve discrepancies through consensus or consultation with another study author (RF, YG). The data charting forms are available in online supplementary appendix 2.

Extracted data will include: 1) Bibliometric details: title, author(s), publication year, journal, 2) Study details: study design, inclusion and exclusion criteria, sample size, sample characteristics, setting, sample size included in analysis, 3) Primary outcome: changes in pediatric ED visits reported during and before the COVID-19 pandemic including numbers, percentages, frequencies, and reasons for change, 4) Secondary outcomes: changes in infant morbidity and mortality details, infant hospitalization, PICU admission, reasons for ED visits and hospital admission and length of ED visit or hospital stay during and before the pandemic.

Step 5: Collating, summarizing, and reporting results

The results from this review will be presented in tables, while pertinent bibliometric details and critical results will be described according to standardized scoping review methodologies. We will collate results exclusively from peer-reviewed journal articles and comment on heterogeneity of the reported results. Extracted data will focus on trends in infant ED usage during and before the COVID-19 pandemic, as well as the impact of the pandemic on infant and neonatal health parameters. Gaps in the literature will be highlighted, and supported by a consultation with a clinical expert in the field. The PRISMA extension for Scoping Reviews (PRISMA-ScR) checklist will be followed to report the results of this scoping review [17].

Step 6: Expert consultation

A consultation with a clinical expert will be sought to strengthen the rigor of the scoping review. Sharing preliminary results with the clinical expert will allow for discussion related to data interpretation, clinical applicability, and dissemination strategies. The clinical expert will provide academic insight beyond what is currently reported in the literature, assist with

For peer review only - http://bmjopen.bmj.com/site/about/guidelines.xhtml

identifying additional sources of information, and inform the clinical relevancy of the scoping review.

Patient and public involvement

The development of this protocol does not require patient or public involvement.

ETHICS AND DISSEMINATION

Ethics approval is not required by the institutional REB as scoping review methodology does not consist of primary data collection. Results from this scoping review will be presented at scientific conferences and be published in a peer-reviewed journal according to the PRISMA-ScR guidelines.

Contributors

YG and SWW conceptualized the study. YG, BO and RF generated and reviewed the inclusion and exclusion criteria. RS developed the search strategy in conjunction with authors YG, RF and BO. BO and MMH designed the data extraction tool. BO drafted the protocol for publication. YG, RF and MMH critically revised the protocol, and all authors approved the manuscript of this protocol for publication. We would like to thank Risa Shorr, medical librarian at The Ottawa Hospital for developing the search strategy and advising on appropriate databases for the review.

Funding

This research is supported by a Canadian Institutes of Health Research Foundation Grant (FDN 148438), a Children's Hospital of Eastern Ontario Research Institute Summer Studentship Award and a University of Ottawa Faculty of Medicine Summer Studentship bursary.

Competing interests

None declared.

Patient and public involvement

Patients and/or public were not involved in the design, conduct, reporting or dissemination strategies of the results of this investigation.

Patient consent for publication

Not required.

For peer review only

I, the Submitting Author has the right to grant and does grant on behalf of all authors of the Work (as defined in the below author licence), an exclusive licence and/or a non-exclusive licence for contributions from authors who are: i) UK Crown employees; ii) where BMJ has agreed a CC-BY licence shall apply, and/or iii) in accordance with the terms applicable for US Federal Government officers or employees acting as part of their official duties; on a worldwide, perpetual, irrevocable, royalty-free basis to BMJ Publishing Group Ltd ("BMJ") its licensees and where the relevant Journal is co-owned by BMJ to the co-owners of the Journal, to publish the Work in BMJ Open and any other BMJ products and to exploit all rights, as set out in our licence.

For peer review only

REFERENCES

1. Ciotti M, Ciccozzi M, Terrinoni A, Jiang WC, Wang CB, Bernardini S. The COVID-19 pandemic. *Critical Reviews in Clinical Laboratory Sciences*. 2020;57(6):365-388. doi:10.1080/10408363.2020.1783198

2. Boserup B, McKenney M, Elkbuli A. The impact of the COVID-19 pandemic on emergency department visits and patient safety in the United States. *The American Journal of Emergency Medicine*. 2020;38(9):1732-1736. doi:10.1016/j.ajem.2020.06.007

3. Hartnett KP. Impact of the COVID-19 Pandemic on Emergency Department Visits — United States, January 1, 2019–May 30, 2020. *MMWR Morb Mortal Wkly Rep*. 2020;69. doi:10.15585/mmwr.mm6923e1

4. Pines JM, Zocchi MS, Black BS, et al. Characterizing pediatric emergency department visits during the COVID-19 pandemic. *Am J Emerg Med*. 2021;41:201-204. doi:10.1016/j.ajem.2020.11.037

5. Dopfer C, Wetzke M, Zychlinsky Scharff A, et al. COVID-19 related reduction in pediatric emergency healthcare utilization – a concerning trend. *BMC Pediatr*. 2020;20. doi:10.1186/s12887-020-02303-6

6. Kruizinga MD, Peeters D, van Veen M, et al. The impact of lockdown on pediatric ED visits and hospital admissions during the COVID19 pandemic: a multicenter analysis and review of the literature. *Eur J Pediatr*. 2021;180(7):2271-2279. doi:10.1007/s00431-021-04015-0

7. Irvine MA, Portales-Casamar E, Goldman RD. An Interrupted Time-Series Analysis of Pediatric Emergency Department Visits During the Coronavirus Disease 2019 Pandemic. *Pediatric Emergency Care*. 2021;37(6):325-328. doi:10.1097/PEC.0000000000002404

8. Finkelstein Y, Maguire B, Zemek R, et al. Effect of the COVID-19 Pandemic on Patient Volumes, Acuity, and Outcomes in Pediatric Emergency Departments: A Nationwide Study. *Pediatr emerg care*. Published online 2021. Accessed June 14, 2021. https://dx.doi.org/10.1097/PEC.0000000000002484

9. Choi DH, Jung JY, Suh D, et al. Impact of the COVID-19 Outbreak on Trends in Emergency Department Utilization in Children: a Multicenter Retrospective Observational Study in Seoul Metropolitan Area, Korea. *J Korean Med Sci*. 2021;36(5). doi:10.3346/jkms.2021.36.e44

10. Aguirre E, Papenburg J, Ouakki M, et al. Comparison of Pandemic and Seasonal Influenza in the Pediatric Emergency Department. *The Pediatric Infectious Disease Journal*. 2011;30(8):633-639. doi:10.1097/INF.0b013e3182103d54

11. Costello BE, Simon HK, Massey R, Hirsh DA. Pandemic H1N1 Influenza in the Pediatric Emergency Department: A Comparison With Previous Seasonal Influenza Outbreaks. *Annals of Emergency Medicine*. 2010;56(6):643-648. doi:10.1016/j.annemergmed.2010.03.001

For peer review only

12. Sachedina N, Donaldson LJ. Paediatric mortality related to pandemic influenza A H1N1 infection in England: an observational population-based study. *The Lancet*. 2010;376(9755):1846-1852. doi:10.1016/S0140-6736(10)61195-6

13. Arksey H, O'Malley L. Scoping studies: towards a methodological framework. *International Journal of Social Research Methodology*. 2005;8(1):19-32. doi:10.1080/1364557032000119616

14. Levac D, Colquhoun H, O'Brien KK. Scoping studies: advancing the methodology. *Implementation Sci*. 2010;5(1):69. doi:10.1186/1748-5908-5-69

15. Peters M, Godfrey C, McInerney P, Munn Z, Trico A, Khalil H. Chapter 11: Scoping Reviews. In: Aromataris E, Munn Z, eds. *JBI Manual for Evidence Synthesis*. JBI; 2020. doi:10.46658/JBIMES-20-12

16. Moher D, Liberati A, Tetzlaff J, Altman DG. Preferred reporting items for systematic reviews and meta-analyses: the PRISMA statement. *BMJ*. 2009;339:b2535. doi:10.1136/bmj.b2535

17. Tricco AC, Lillie E, Zarin W, et al. PRISMA Extension for Scoping Reviews (PRISMA-ScR): Checklist and Explanation. *Ann Intern Med*. 2018;169(7):467-473. doi:10.7326/M18-0850

For peer review only

For peer review only - http://bmjopen.bmj.com/site/about/guidelines.xhtml

Appendix 1: Search Strategies

1.1 Search Strategy COVID-19 Emergency Department Visits

Embase Classic+Embase <1947 to 2021 June 22>
Ovid MEDLINE(R) ALL <1946 to June 22, 2021>

1 COVID-19/ 89102
2 (exp coronavirus/ or coronavirus*.mp. or corona virus*.mp.) and (wuhan or beijing or shanghai or hubei).mp. 12025
3 ((coronavirus* or corona virus* or coronavirus* or coronaviridae or coronaviridae or betacoronavirus*) adj3 ("19" or "2019")).tw. 73359
4 covid.tw,kw. 257302
5 covid19.tw,kw. or covid 19.kw. 116191
6 sars cov 2.tw,kw. 93714
7 (ncov or n cov).tw,kw. 3527
8 (novel coronavirus* or novel corona virus*).tw,kw. 18402
9 (CoV 2 or CoV2 or sarscov2 or 2019nCoV or novel CoV or wuhan virus*).tw,kw. 90445
10 (Coronavirus Infections/ or Severe Acute Respiratory Syndrome/) and (Pandemics/ or pandemic*.tw,kf.) 50801
11 or/1-10293685
12 exp Emergency Service, Hospital/ 91695
13 emergency department*.tw,kf. 260553
14 (emergency room* or emergency visit* or ed visit*).tw,kf. or emergenc*.ti. 342084
15 or/12-14 519228
16 11 and 15 9756
17 infant/ or infant, newborn/ 2329710
18 (infant* or baby or babies or neonat* or newborn*).tw,kf. 1782687
19 17 or 18 3049171
20 16 and 19 373
21 20 use medall 197 Medline
22 coronavirus disease 2019/ 210486
23 (Coronavirinae/ or coronavirus*.mp. or corona virus*.mp.) and (wuhan or beijing or shanghai or hubei).mp. 11723
24 ((coronavirus* or corona virus* or coronavirus* or coronaviridae or coronaviridae or betacoronavirus*) adj3 ("19" or "2019")).tw. 73359
25 (covid or covid19).tw.253757
26 sars cov 2.tw. 81000
27 (ncov or n cov).tw. 3072
28 (novel coronavirus* or novel corona virus*).tw. 17339
29 (CoV 2 or CoV2 or sarscov2 or 2019nCoV or novel CoV or wuhan virus).tw. 83852
30 (coronavirus infection/ or severe acute respiratory syndrome/) and (pandemic/ or pandemic*.tw.) 50877
31 limit 30 to yr="2019 -Current" 49777
32 or/22-29,31 300886
33 exp infant/ 2383856
34 (infant* or baby or babies or neonat* or newborn*).tw. 1741143

For peer review only

35 33 or 34 3054401
36 32 and 35 7549
37 emergency ward/ 240135
38 emergency department*.tw. 259493
39 (emergency room* or emergency visit* or ed visit*).tw. or emergenc*.ti. 342000
40 emergency health service/ 151957
41 or/37-40 635540
42 36 and 41 430
43 42 use emczd 233 Embase
44 21 or 43 430
45 remove duplicates from 44 320
46 45 use medall 194
47 45 use emczd 126

Web of Science – June 23, 2021
7
76
#6 AND #5
Indexes=SCI-EXPANDED, CPCI-S, ESCI Timespan=1900-2021

6
765,057
TOPIC:
(infant* or baby or babies or neonat* or newborn*)
Indexes=SCI-EXPANDED, CPCI-S, ESCI Timespan=1900-2021

5
5,159
#4 AND #3
Indexes=SCI-EXPANDED, CPCI-S, ESCI Timespan=1900-2021

4
258,186
TS=(emergency room* or emergency visit* or ed visit* or emergency department*) OR TI=(emergenc*)
OR TS=(emergency service*)
Indexes=SCI-EXPANDED, CPCI-S, ESCI Timespan=1900-2021

3
132,967
#2 OR #1
Indexes=SCI-EXPANDED, CPCI-S, ESCI Timespan=1900-2021

2
49,511
TOPIC: (sars cov 2 OR sars cov2) OR TOPIC: (ncov or n cov)
OR TOPIC: (novel coronavirus or novel corona virus)
Indexes=SCI-EXPANDED, CPCI-S, ESCI Timespan=1900-2021

1

For peer review only

121,251
TOPIC: (covid OR covid19 or covid2019)
Indexes=SCI-EXPANDED, CPCI-S, ESCI Timespan=1900-2021

Cinahl - June 23, 2021

Wednesday, June 23, 2021 6:25:31 PM

#	Query	Results
S1	(MH "COVID-19")	16,649
S2	(MH "COVID-19 Pandemic")	14,878
S3	(MH "SARS-CoV-2")	370
S4	TI covid* OR AB covid* OR TI sars cov 2 OR AB sars cov 2 OR TI novel coronavirus OR AB novel coronavirus OR TI wuhan virus OR AB wuhan virus OR TI 2019nCoV OR AB 2019nCoV	46,116
S5	TI (CoV 2 or CoV2 or sarscov2) OR AB (CoV 2 or CoV2 or sarscov2)	200
S6	S1 OR S2 OR S3 OR S4 OR S5	54,533
S7	(MH "Emergency Service+")	63,535
S8	emergency department*	73,679
S9	(emergency room* or emergency visit* or ed visit*)	46,343
S10	TI emergenc*	66,600
S11	(MH "Emergency Medical Services")	26,978
S12	(S7 OR S8 OR S9 OR S10 OR S11)	149,918
S13	(S6 AND S12)	2,014
S14	(MH "Infant+")	265,404
S15	TI ((infant* or baby or babies or neonat* or newborn*)) OR AB ((infant* or baby or babies or neonat* or newborn*))	193,442
S16	S14 OR S15	345,326
S17	(S13 AND S16)	58

For peer review only - http://bmjopen.bmj.com/site/about/guidelines.xhtml

1.2 Search Strategy COVID-19 Neonatal Mortality

Embase Classic+Embase <1947 to 2021 June 22>
Ovid MEDLINE(R) ALL <1946 to June 22, 2021>

1 COVID-19/ 89102
2 (exp coronavirus/ or coronavirus*.mp. or corona virus*.mp.) and (wuhan or beijing or shanghai or hubei).mp. 12025
3 ((coronavirus* or corona virus* or coronavirus* or coronaviridae or coronaviridae or betacoronavirus*) adj3 ("19" or "2019")).tw. 73359
4 covid.tw,kw. 257302
5 covid19.tw,kw. or covid 19.kw. 116191
6 sars cov 2.tw,kw. 93714
7 (ncov or n cov).tw,kw. 3527
8 (novel coronavirus* or novel corona virus*).tw,kw. 18402
9 (CoV 2 or CoV2 or sarscov2 or 2019nCoV or novel CoV or wuhan virus*).tw,kw. 90445
10 (Coronavirus Infections/ or Severe Acute Respiratory Syndrome/) and (Pandemics/ or pandemic*.tw,kf.) 50801
11 or/1-10293685
12 exp Infant Mortality/ 56249
13 exp infant death/ or perinatal death/ 41371
14 ((infant* or baby or babies or neonat* or newborn* or perinat*) adj3 (death* or mortalit*)).tw,kf. 134966
15 12 or 13 or 14 174602
16 11 and 15 436
17 16 use medall 180 Medline
18 coronavirus disease 2019/ 210486
19 (Coronavirinae/ or coronavirus*.mp. or corona virus*.mp.) and (wuhan or beijing or shanghai or hubei).mp. 11723
20 ((coronavirus* or corona virus* or coronavirus* or coronaviridae or coronaviridae or betacoronavirus*) adj3 ("19" or "2019")).tw. 73359
21 (covid or covid19).tw.253757
22 sars cov 2.tw. 81000
23 (ncov or n cov).tw. 3072
24 (novel coronavirus* or novel corona virus*).tw. 17339
25 (CoV 2 or CoV2 or sarscov2 or 2019nCoV or novel CoV or wuhan virus).tw. 83852
26 (coronavirus infection/ or severe acute respiratory syndrome/) and (pandemic/ or pandemic*.tw.) 50877
27 limit 26 to yr="2019 -Current" 49777
28 or/18-25,27 300886
29 infant mortality/ 54661
30 newborn mortality/ 15392
31 perinatal mortality/ 17344
32 perinatal death/ 6323
33 ((infant* or baby or babies or neonat* or newborn*) adj2 (death* or mortalit*)).tw. 85685
34 or/29-33 141402
35 28 and 34 359

For peer review only

For peer review only - http://bmjopen.bmj.com/site/about/guidelines.xhtml

**36**	**35 use emczd**	**224**	**Embase**
37	17 or 36	404	
38	remove duplicates from 37	269	
39	38 use medall	177	
40	38 use emczd	92	

Web of Science – June 23, 2021

# 5	160	#4 AND #3<br>*Indexes=SCI-EXPANDED, CPCI-S, ESCI Timespan=1900-2021*
# 4	**46,989**	**TOPIC:** ((infant* or baby or babies or neonat* or newborn*) NEAR/5 (death* or mortalit*))<br>*Indexes=SCI-EXPANDED, CPCI-S, ESCI Timespan=1900-2021*
# 3	**132,967**	#2 OR #1<br>*Indexes=SCI-EXPANDED, CPCI-S, ESCI Timespan=1900-2021*
# 2	**49,511**	**TOPIC:** (sars cov 2 OR sars cov2) *OR* **TOPIC:** (ncov or n cov) *OR* **TOPIC:** (novel coronavirus or novel corona virus)<br>*Indexes=SCI-EXPANDED, CPCI-S, ESCI Timespan=1900-2021*
# 1	**121,251**	**TOPIC:** (covid OR covid19 or covid2019)<br>*Indexes=SCI-EXPANDED, CPCI-S, ESCI Timespan=1900-2021*

Cinahl – June 23, 2021

**#**	**Query**	**Results**
S1	(MH "COVID-19")	16,649
S2	(MH "COVID-19 Pandemic")	14,878
S3	(MH "SARS-CoV-2")	370
S4	TI covid* OR AB covid* OR TI sars cov 2 OR AB sars cov 2 OR TI novel coronavirus OR AB novel coronavirus OR TI wuhan virus OR AB wuhan virus OR TI 2019nCoV OR AB 2019nCoV	46,116
S5	TI (CoV 2 or CoV2 or sarscov2) OR AB (CoV 2 or CoV2 or sarscov2)	200
S6	S1 OR S2 OR S3 OR S4 OR S5	54,533
S7	(MH "Infant Mortality")	9,417
S8	(MH "Infant Death")	860

For peer review only

S9	(MH "Perinatal Death")	8,356
S10	TI (((infant* or baby or babies or neonat* or newborn* or perinat*) N3 (death* or mortalit*))) OR AB (((infant* or baby or babies or neonat* or newborn* or perinat*) N3 (death* or mortalit*)))	17,335
S11	(S7 OR S8 OR S9 OR S10)	28,136
S12	S6 AND S11	**89**

For peer review only

For peer review only - http://bmjopen.bmj.com/site/about/guidelines.xhtml

Appendix 2: Data Charting Form

Data charting framework

Main category	Subcategory	Description
**Titles**		
**Authors**		
**Year of publication**		
**Study Journal**		
**Study Citation Details**		
**Study objective(s)**		Describe the stated objectives of the studies included in the review
**Study Design**		Specify the types of studies included in the review
**Study Setting**		Specify the geographical areas covered by the studies included in the review
**Study Population/Sample size**		Describe the study population and specify the number of participants included in each studies
**Eligibility Criteria**		Specify the inclusion and exclusion criteria of each studies included in the review
**Study period**		Specify the time period of data collection of each studies included in the review
**Reported primary outcomes (neonates <28 days and infants <1 year-old)**	Frequency of ED visits	Specify the frequency of ED visits before and during the COVID-19 pandemic
	Main Reason(s) for ED visits	Describe the main reasons for ED visits before and during the COVID-19 pandemic
**Reported secondary outcomes (neonates <28 days and infants <1 year-old)**	Change in mortality	Specify if there was an increase or decrease of neonatal/infant mortality during the COVID-19 pandemic compared to pre-pandemic
	Causes of mortality	Describe the main causes of mortality before and during the COVID-19 pandemic
	ED visit length	Specify the ED visit length (in hours) during the COVID-19 pandemic compared to pre-pandemic
	NICU/PICU admission	Specify the NICU/PICU admission rate before and during the COVID-19 pandemic
	Reasons for NICU/PICU admission	Describe the main reasons of NICU/PICU admission before and during the COVID-19 pandemic
	NICU/PICU length of stay	Specify the NICU/PICU length of stay (in days) during the COVID-19 pandemic compared to pre-pandemic
	Infant hospitalization	Specify the infant hospitalization rate before and during the COVID-19 pandemic
	Reasons for infant hospitalization	Describe the main reasons of infant hospitalization before and during the COVID-19 pandemic

For peer review only - http://bmjopen.bmj.com/site/about/guidelines.xhtml

BMJ Open

Impact of COVID-19 pandemic on emergency department visits and infant health: a scoping review protocol

Journal:	*BMJ Open*
Manuscript ID	bmjopen-2022-061778.R1
Article Type:	Protocol
Date Submitted by the Author:	19-May-2022
Complete List of Authors:	Osborne, Brenden; Children's Hospital of Eastern Ontario Research Institute; Ottawa Hospital Research Institute<br>Moorjani-Houle, Mélika; Ottawa Hospital Research Institute; University of Ottawa<br>Fakhraei, Romina; Ottawa Hospital Research Institute; University of Ottawa<br>Walker, Mark; Ottawa Hospital Research Institute; University of Ottawa, Department of Obstetrics and Gynecology<br>Wen, Shi Wu; Ottawa Hospital Research Institute; University of Ottawa<br>Guo, Yanfang; Children's Hospital of Eastern Ontario, BORN Ontario; University of Ottawa, School of Epidemiology and Public Health
**Primary Subject Heading**:	Epidemiology
Secondary Subject Heading:	Paediatrics, Emergency medicine
Keywords:	COVID-19, Paediatric A&E and ambulatory care < PAEDIATRICS, EPIDEMIOLOGY, Community child health < PAEDIATRICS

SCHOLARONE™
Manuscripts

Impact of COVID-19 pandemic on emergency department visits and infant health: a scoping review protocol

Authors:
Osborne, Brenden[1,2], Moorjani-Houle, Mélika[2,3], Fakhraei, Romina[2,4], Walker, Mark[2,4,5], Wen, Shi-Wu[2,4,5], Guo, Yanfang,[1,2,4,6*]

Affiliations:
1. Children's Hospital of Eastern Ontario Research Institute, Ottawa, Ontario, Canada;
2. OMNI Research Group, Clinical Epidemiology Program, Ottawa Hospital Research Institute, Ottawa, Ontario, Canada;
3. Faculty of Medicine, University of Ottawa, Ottawa, Canada;
4. School of Epidemiology and Public Health, University of Ottawa, Ottawa, Ontario, Canada;
5. Department of Obstetrics and Gynecology, University of Ottawa, Ottawa, Ontario, Canada;
6. Better Outcomes Registry & Network Ontario, Ottawa, Ontario, Canada

E-mail Addresses: bosborne@toh.ca (B. Osborne), mmoorjani@ohri.ca (M. M-Houle), rfakhraei@ohri.ca (R. Fakhraei), mwalker@toh.ca (M. Walker), swwen@ohri.ca (SW. Wen), yguo@bornontario.ca (Y. Guo)

***Corresponding Author:**

Dr. Yanfang Guo
Better Outcomes Registry & Network Ontario
CHEO Research Institute – Centre for Practice Changing Research Building
401 Smyth Road, Ottawa, ON K1H 8L6
T: 613-737-8899 (x 73840)
Email: yguo@bornontario.ca

For peer review only

For peer review only - http://bmjopen.bmj.com/site/about/guidelines.xhtml

ABSTRACT

Introduction

The novel SARS-CoV-2 pandemic has provided a set of unique challenges for pediatric patients requiring emergency care across the globe. Reduction in pediatric emergency department (ED) usage during the COVID-19 pandemic has been widely reported, but no studies to date have consolidated and described what ramifications these reductions may have on neonatal and infant health. This scoping review aims to characterize the impact of the COVID-19 pandemic on infant emergency department visits and neonatal and infant health.

Methods and analysis

A comprehensive literature search will be conducted from March 2020 to June 2021 using the following databases: Embase (OVID), Web of Science (Clarivate Analytics), Medline (Ovid) and CINAHL (EBSCOhost). This scoping review will use a five-step framework to guide the selection, extraction and analysis of data from eligible studies, with an additional sixth step for clinical consultation. Studies in English reporting the effect of the COVID-19 pandemic on infant ED visits, as well as neonatal and infant health will be included for screening. Key findings will be reported according to the Preferred Reporting Items for Systematic Reviews and Meta-Analysis Extension for Scoping Reviews.

Ethics and dissemination

Research ethics board approval will not be required due to the nature of study design. The results of this scoping review will be disseminated through publication in a peer-reviewed journal and presentation at academic conferences.

Strengths and limitations of this study

- Peer-reviewed framework for scoping reviews (Arksey & O'Malley) was used to design this study.
- Selected studies will compare data prior to and during the COVID-19 pandemic to assess trend differences.
- A clinical expert will be consulted to inform the applicability of study results.
- Availability of relevant data in selected studies may vary per site.

For peer review only

INTRODUCTION

In March 2020 the World Health Organization declared the novel Coronavirus (COVID-19) outbreak a global pandemic, which continues to affect millions globally [1]. As individuals restrict their movements, undergo mandatory social distancing, and work from home, hospitals around the globe are observing a reduced patient load in their emergency departments (ED) [2]. The Centre for Disease Control and Prevention reports a 42% decrease in ED visits in the United States based on weekly means, dropping from 2.1 million per week pre-pandemic to 1.2 million during the pandemic, with the steepest decrease reported in the pediatric patient population [3].

Several studies report a steep decrease in pediatric ED visits since the COVID-19 pandemic began [4–7]. A Canadian study of eleven pediatric centers reported a decrease in ED visits by 58% during the pandemic, compared to estimated rates [8]. The literature highlights that reduced ED usage may be a result of increased lockdown measures, social distancing, and fear of contracting SARS-CoV-2 [5]. Data from Korea highlights the association between increasing government restrictions and a reduction in the number of monthly ED visits [9].

Previous work detailing the effect of the H1N1 pandemic on infant health, morbidity, and mortality may offer insight regarding potential effects of SARS-CoV-2 infection in this population. Studies report delays in presentation to the ED during the H1N1 pandemic (2.8 ± 2.3 days) as well an increase in pediatric intensive care unit (PICU) admissions during this time compared to years prior (0.3% vs 0.1%; 95% CI 0.05%, 0.4%) [10,11]. Children younger than two years of age with a confirmed H1N1 diagnosis were reported to have an increased risk of hospitalization during this period (RR 3.3; 95% CI 1.80, 6.05) [10]. One population-based study in England reported the highest case-fatality rate in their infant (<1year) population, with a fatality rate of 151 per 100,000 cases of H1N1[12].

To date, most studies have focused on collecting or retrospectively analyzing primary data to quantify the reduction in pediatric ED usage during the COVID-19 pandemic. The literature is lacking consensus on the implications of these reductions on infant health outside the context of a confirmed COVID-19 diagnosis, in the general infant population. Given what is known about previous pandemics precipitating adverse effects on infant health [10–12], further knowledge synthesis is required to address the novel situation created by the COVID-19 pandemic. Our objective is to conduct a scoping review to characterize the effect of, and to better understand the impact of the COVID-19 pandemic on infant emergency department visits and secondarily, neonatal and infant health.

METHODS AND ANALYSIS

This scoping review will be conducted according to the methodological framework developed by Arksey and O'Malley and refined by Levac *et al* and the Joanna Briggs Institute (JBI) [13–15]. The steps outline by these frameworks are: (1) identification of the research question, (2) identification of relevant studies, (3) selection of studies, (4) charting the data, and (5) collating, summarizing, and reporting results. As recommended by Arksey and O'Malley [13], we included an additional consultation step with a clinical expert, to find additional sources of information and inform the clinical relevance and value of this review.

For peer review only - http://bmjopen.bmj.com/site/about/guidelines.xhtml

Step 1: Identifying the research question

To help identify the main concepts of the primary review question, the Population/Concepts/Context (PCC) framework is suggested by the JBI [15]. The specific PCC framework for this study is presented in **Table 1**. The primary research question for this review is: what is the current evidence reporting the effect of the COVID-19 pandemic on infant emergency department visits, neonatal, and infant health?

Table 1 Population-Concept-Context

**Population**	All peer-reviewed journal articles including neonates (<28 days) and infants (<1 year) will be included.
**Concepts**	Literature reporting on the frequency/rate and main reasons for neonate/infant ED visits during and before the COVID-19 pandemic will be reviewed. Literature reporting on infant outcomes; infant mortality including neonatal death (< 28 days) and infant death (< 1 year of age), main reasons for infant mortality, infant and neonate hospitalization, main reasons for hospital admission, pediatric or neonatal intensive care unit admission, high dependency unit admission and length of ED visit and/or hospital stay will also be reviewed.
**Context**	The context will be hospital emergency departments. The time-frame is before and during the COVID-19 pandemic (March 2020 and onwards). There will be no restrictions on geographical location.

ED, emergency department.

Step 2: Identifying relevant studies

Our search strategy and database choice will be developed in conjunction with, and refined by a trained medical librarian. Four online databases will be searched, including: Embase (OVID), Web of Science (Clarivate Analytics), Medline (Ovid) and CINAHL (EBSCOhost). Key search terms were developed to capture literature related to the effect of the COVID-19 pandemic on infant ED visits, as well as neonatal and infant health. Truncation and Boolean were used to narrow, widen and combine search parameters as necessary. The finalized search strategies are available in online supplementary appendix 1. The initial literature search will be carried out July 15th, 2022 and the study will be completed September 15th, 2022.

We agreed to the following eligibility criteria for the initial search:

- Type of publication: journal articles.
- Time frame: during the COVID-19 pandemic (March 2020 onwards).
- Study population: neonates (<28 days of age) and infants (< 1 year of age) presenting to the ED for medical attention.
- Study designs: Analytic epidemiological observational study designs (i.e. cohort studies, case-control studies or cross-sectional design studies), analytical ecological studies (i.e. time series studies), and systematic reviews with or without meta-analyses.

For peer review only - http://bmjopen.bmj.com/site/about/guidelines.xhtml

We will exclude: case reports and series, editorials, commentaries, letters to the editor, abstracts, conference proceedings, book chapters, narrative reviews, pre-print literature, non-English studies and any studies that do not compare outcome data collected during the pandemic with a time period prior to the pandemic.

Step 3: Study selection

Records from electronic database searches will be imported into Microsoft Excel to eliminate duplicates. Two reviewers (BO, MMH) will independently screen titles and abstracts to determine study eligibility based on predefined inclusion and exclusion criteria. A second screen of the article in full-text will be performed by two independent reviewers (BO, MMH) to ensure that studies fully meet inclusion criteria and report relevant data. Any discrepancies will be resolved by consensus or through a third reviewer (RF). The results of the screening process will be displayed by a Preferred Reporting Items for Systematic Reviews and Meta-Analyses (PRISMA) flow diagram [16].

Step 4: Charting the data

Data will be extracted into standardized forms in Excel by two reviewers (BO, MMH) and checked for accuracy and completeness by a third reviewer (RF). Whenever necessary, the authors of the original study will be contacted for additional information and clarification of the data. Reviewers will resolve discrepancies through consensus or consultation with another study author (RF, YG). The data charting forms are available in online supplementary appendix 2.

Extracted data will include: 1) Bibliometric details: title, author(s), publication year, journal, 2) Study details: study design, inclusion and exclusion criteria, sample size, sample characteristics, setting, sample size included in analysis, study period 3) Primary outcome: changes in pediatric ED visits reported during and before the COVID-19 pandemic including numbers, percentages, frequencies, and reasons for change, 4) Secondary outcomes: changes in infant morbidity and mortality details, neonatal intensive care unit (NICU) admission, PICU or high-dependency unit (HDU) admission, infant hospitalizations, reasons for ED visits or hospital admission and length of ED visit or hospital stay during and before the pandemic.

Step 5: Collating, summarizing, and reporting results

The results from this review will be presented in tables, while pertinent bibliometric details and critical results will be described according to standardized scoping review methodologies. We will collate results exclusively from peer-reviewed journal articles and comment on heterogeneity of the reported results. Extracted data will focus on trends in infant ED usage during and before the COVID-19 pandemic, as well as the impact of the pandemic on infant and neonatal health parameters. Gaps in the literature will be highlighted, and supported by a consultation with a clinical expert in the field. The PRISMA extension for Scoping Reviews (PRISMA-ScR) checklist will be followed to report the results of this scoping review [17].

Step 6: Expert consultation

A consultation with a clinical expert will be sought to strengthen the rigor of the scoping review. Sharing preliminary results with the clinical expert will allow for discussion related to data interpretation, clinical applicability, and dissemination strategies. The clinical expert will provide academic insight beyond what is currently reported in the literature, assist with

For peer review only - http://bmjopen.bmj.com/site/about/guidelines.xhtml

identifying additional sources of information, and inform the clinical relevancy of the scoping review.

Patient and public involvement

Patients and the public were not involved in the development of this protocol.

ETHICS AND DISSEMINATION

Ethics approval is not required by the institutional REB as scoping review methodology does not consist of primary data collection. Results from this scoping review will be presented at scientific conferences and be published in a peer-reviewed journal according to the PRISMA-ScR guidelines.

Contributors

YG, SWW and MW conceptualized the study. YG, BO and RF generated and reviewed the inclusion and exclusion criteria. RS developed the search strategy in conjunction with authors YG, RF and BO. BO and MMH designed the data extraction tool. BO and MMH drafted the protocol for publication. All authors critically revised and approved the manuscript of this protocol for publication. We would like to thank Risa Shorr, medical librarian at The Ottawa Hospital for developing the search strategy and advising on appropriate databases for the review.

Funding

This research is supported by a Canadian Institutes of Health Research Foundation Grant (FDN 148438), a Children's Hospital of Eastern Ontario Research Institute Summer Studentship Award and a University of Ottawa Faculty of Medicine Summer Studentship bursary.

Competing interests

None declared.

Patient consent for publication

Not required.

For peer review only

I, the Submitting Author has the right to grant and does grant on behalf of all authors of the Work (as defined in the below author licence), an exclusive licence and/or a non-exclusive licence for contributions from authors who are: i) UK Crown employees; ii) where BMJ has agreed a CC-BY licence shall apply, and/or iii) in accordance with the terms applicable for US Federal Government officers or employees acting as part of their official duties; on a worldwide, perpetual, irrevocable, royalty-free basis to BMJ Publishing Group Ltd ("BMJ") its licensees and where the relevant Journal is co-owned by BMJ to the co-owners of the Journal, to publish the Work in BMJ Open and any other BMJ products and to exploit all rights, as set out in our licence.

For peer review only

REFERENCES

1. Ciotti M, Ciccozzi M, Terrinoni A, Jiang WC, Wang CB, Bernardini S. The COVID-19 pandemic. *Critical Reviews in Clinical Laboratory Sciences*. 2020;57(6):365-388. doi:10.1080/10408363.2020.1783198

2. Boserup B, McKenney M, Elkbuli A. The impact of the COVID-19 pandemic on emergency department visits and patient safety in the United States. *The American Journal of Emergency Medicine*. 2020;38(9):1732-1736. doi:10.1016/j.ajem.2020.06.007

3. Hartnett KP. Impact of the COVID-19 Pandemic on Emergency Department Visits — United States, January 1, 2019–May 30, 2020. *MMWR Morb Mortal Wkly Rep*. 2020;69. doi:10.15585/mmwr.mm6923e1

4. Pines JM, Zocchi MS, Black BS, et al. Characterizing pediatric emergency department visits during the COVID-19 pandemic. *Am J Emerg Med*. 2021;41:201-204. doi:10.1016/j.ajem.2020.11.037

5. Dopfer C, Wetzke M, Zychlinsky Scharff A, et al. COVID-19 related reduction in pediatric emergency healthcare utilization – a concerning trend. *BMC Pediatr*. 2020;20. doi:10.1186/s12887-020-02303-6

6. Kruizinga MD, Peeters D, van Veen M, et al. The impact of lockdown on pediatric ED visits and hospital admissions during the COVID19 pandemic: a multicenter analysis and review of the literature. *Eur J Pediatr*. 2021;180(7):2271-2279. doi:10.1007/s00431-021-04015-0

7. Irvine MA, Portales-Casamar E, Goldman RD. An Interrupted Time-Series Analysis of Pediatric Emergency Department Visits During the Coronavirus Disease 2019 Pandemic. *Pediatric Emergency Care*. 2021;37(6):325-328. doi:10.1097/PEC.0000000000002404

8. Finkelstein Y, Maguire B, Zemek R, et al. Effect of the COVID-19 Pandemic on Patient Volumes, Acuity, and Outcomes in Pediatric Emergency Departments: A Nationwide Study. *Pediatr emerg care*. Published online 2021. Accessed June 14, 2021. https://dx.doi.org/10.1097/PEC.0000000000002484

9. Choi DH, Jung JY, Suh D, et al. Impact of the COVID-19 Outbreak on Trends in Emergency Department Utilization in Children: a Multicenter Retrospective Observational Study in Seoul Metropolitan Area, Korea. *J Korean Med Sci*. 2021;36(5). doi:10.3346/jkms.2021.36.e44

10. Aguirre E, Papenburg J, Ouakki M, et al. Comparison of Pandemic and Seasonal Influenza in the Pediatric Emergency Department. *The Pediatric Infectious Disease Journal*. 2011;30(8):633-639. doi:10.1097/INF.0b013e3182103d54

11. Costello BE, Simon HK, Massey R, Hirsh DA. Pandemic H1N1 Influenza in the Pediatric Emergency Department: A Comparison With Previous Seasonal Influenza Outbreaks. *Annals of Emergency Medicine*. 2010;56(6):643-648. doi:10.1016/j.annemergmed.2010.03.001

For peer review only - http://bmjopen.bmj.com/site/about/guidelines.xhtml

12. Sachedina N, Donaldson LJ. Paediatric mortality related to pandemic influenza A H1N1 infection in England: an observational population-based study. *The Lancet*. 2010;376(9755):1846-1852. doi:10.1016/S0140-6736(10)61195-6

13. Arksey H, O'Malley L. Scoping studies: towards a methodological framework. *International Journal of Social Research Methodology*. 2005;8(1):19-32. doi:10.1080/1364557032000119616

14. Levac D, Colquhoun H, O'Brien KK. Scoping studies: advancing the methodology. *Implementation Sci*. 2010;5(1):69. doi:10.1186/1748-5908-5-69

15. Peters M, Godfrey C, McInerney P, Munn Z, Trico A, Khalil H. Chapter 11: Scoping Reviews. In: Aromataris E, Munn Z, eds. *JBI Manual for Evidence Synthesis*. JBI; 2020. doi:10.46658/JBIMES-20-12

16. Moher D, Liberati A, Tetzlaff J, Altman DG. Preferred reporting items for systematic reviews and meta-analyses: the PRISMA statement. *BMJ*. 2009;339:b2535. doi:10.1136/bmj.b2535

17. Tricco AC, Lillie E, Zarin W, et al. PRISMA Extension for Scoping Reviews (PRISMA-ScR): Checklist and Explanation. *Ann Intern Med*. 2018;169(7):467-473. doi:10.7326/M18-0850

For peer review only

Appendix 1: Search Strategies

1.1 Search Strategy COVID-19 Emergency Department Visits

Embase Classic+Embase <1947 to 2021 June 22>
Ovid MEDLINE(R) ALL <1946 to June 22, 2021>

1 COVID-19/
2 (exp coronavirus/ or coronavirus*.mp. or corona virus*.mp.) and (wuhan or beijing or shanghai or hubei).mp.
3 ((coronavirus* or corona virus* or coronavirus* or coronaviridae or coronaviridae or betacoronavirus*) adj3 ("19" or "2019")).tw.
4 covid.tw,kw.
5 covid19.tw,kw. or covid 19.kw.
6 sars cov 2.tw,kw.
7 (ncov or n cov).tw,kw.
8 (novel coronavirus* or novel corona virus*).tw,kw.
9 (CoV 2 or CoV2 or sarscov2 or 2019nCoV or novel CoV or wuhan virus*).tw,kw.
10 (Coronavirus Infections/ or Severe Acute Respiratory Syndrome/) and (Pandemics/ or pandemic*.tw,kf.)
11 or/1-
12 exp Emergency Service, Hospital/
13 emergency department*.tw,kf.
14 (emergency room* or emergency visit* or ed visit*).tw,kf. or emergenc*.ti.
15 or/12-14
16 11 and 15
17 infant/ or infant, newborn/
18 (infant* or baby or babies or neonat* or newborn*).tw,kf.
19 17 or 18
20 16 and 19
21 20 use medall Medline
22 coronavirus disease 2019/
23 (Coronavirinae/ or coronavirus*.mp. or corona virus*.mp.) and (wuhan or beijing or shanghai or hubei).mp.
24 ((coronavirus* or corona virus* or coronavirus* or coronaviridae or coronaviridae or betacoronavirus*) adj3 ("19" or "2019")).tw.
25 (covid or covid19).tw.
26 sars cov 2.tw.
27 (ncov or n cov).tw.
28 (novel coronavirus* or novel corona virus*).tw.
29 (CoV 2 or CoV2 or sarscov2 or 2019nCoV or novel CoV or wuhan virus).tw.
30 (coronavirus infection/ or severe acute respiratory syndrome/) and (pandemic/ or pandemic*.tw.)
31 limit 30 to yr="2019 -Current"
32 or/22-29,31
33 exp infant/

For peer review only

34 (infant* or baby or babies or neonat* or newborn*).tw.
35 33 or 34
36 32 and 35
37 emergency ward/
38 emergency department*.tw.
39 (emergency room* or emergency visit* or ed visit*).tw. or emergenc*.ti.
40 emergency health service/
41 or/37-40
42 36 and 41
43 42 use emczd Embase
44 21 or 43
45 remove duplicates from 44
46 45 use medall
47 45 use emczd

Web of Science – June 23, 2021
7
#6 AND #5
Indexes=SCI-EXPANDED, CPCI-S, ESCI Timespan=1900-2021

6
TOPIC:
(infant* or baby or babies or neonat* or newborn*)
Indexes=SCI-EXPANDED, CPCI-S, ESCI Timespan=1900-2021

5
#4 AND #3
Indexes=SCI-EXPANDED, CPCI-S, ESCI Timespan=1900-2021

# 4
TS=(emergency room* or emergency visit* or ed visit* or emergency department*) OR TI=(emergenc*)
OR TS=(emergency service*)
Indexes=SCI-EXPANDED, CPCI-S, ESCI Timespan=1900-2021

3
#2 OR #1
Indexes=SCI-EXPANDED, CPCI-S, ESCI Timespan=1900-2021

2
TOPIC: (sars cov 2 OR sars cov2) OR TOPIC: (ncov or n cov)
OR TOPIC: (novel coronavirus or novel corona virus)
Indexes=SCI-EXPANDED, CPCI-S, ESCI Timespan=1900-2021

1
TOPIC: (covid OR covid19 or covid2019)

For peer review only

Indexes=SCI-EXPANDED, CPCI-S, ESCI Timespan=1900-2021

Cinahl - June 23, 2021

Wednesday, June 23, 2021 6:25:31 PM

#	Query
S1	(MH "COVID-19")
S2	(MH "COVID-19 Pandemic")
S3	(MH "SARS-CoV-2")
S4	TI covid* OR AB covid* OR TI sars cov 2 OR AB sars cov 2 OR TI novel coronavirus OR AB novel coronavirus OR TI wuhan virus OR AB wuhan virus OR TI 2019nCoV OR AB 2019nCoV
S5	TI (CoV 2 or CoV2 or sarscov2) OR AB (CoV 2 or CoV2 or sarscov2)
S6	S1 OR S2 OR S3 OR S4 OR S5
S7	(MH "Emergency Service+")
S8	emergency department*
S9	(emergency room* or emergency visit* or ed visit*)
S10	TI emergenc*
S11	(MH "Emergency Medical Services")
S12	(S7 OR S8 OR S9 OR S10 OR S11)
S13	(S6 AND S12)
S14	(MH "Infant+")
S15	TI ((infant* or baby or babies or neonat* or newborn*)) OR AB ((infant* or baby or babies or neonat* or newborn*))
S16	S14 OR S15
S17	(S13 AND S16)

For peer review only

1.2 Search Strategy COVID-19 Neonatal Mortality

Embase Classic+Embase <1947 to 2021 June 22>
Ovid MEDLINE(R) ALL <1946 to June 22, 2021>

1 COVID-19/
2 (exp coronavirus/ or coronavirus*.mp. or corona virus*.mp.) and (wuhan or beijing or shanghai or hubei).mp.
3 ((coronavirus* or corona virus* or coronavirus* or coronaviridae or coronaviridae or betacoronavirus*) adj3 ("19" or "2019")).tw.
4 covid.tw,kw.
5 covid19.tw,kw. or covid 19.kw.
6 sars cov 2.tw,kw.
7 (ncov or n cov).tw,kw.
8 (novel coronavirus* or novel corona virus*).tw,kw.
9 (CoV 2 or CoV2 or sarscov2 or 2019nCoV or novel CoV or wuhan virus*).tw,kw.
10 (Coronavirus Infections/ or Severe Acute Respiratory Syndrome/) and (Pandemics/ or pandemic*.tw,kf.)
11 or/1
12 exp Infant Mortality/
13 exp infant death/ or perinatal death/
14 ((infant* or baby or babies or neonat* or newborn* or perinat*) adj3 (death* or mortalit*)).tw,kf.
15 12 or 13 or 14
16 11 and 15
17 16 use medall Medline
18 coronavirus disease 2019/
19 (Coronavirinae/ or coronavirus*.mp. or corona virus*.mp.) and (wuhan or beijing or shanghai or hubei).mp.
20 ((coronavirus* or corona virus* or coronavirus* or coronaviridae or coronaviridae or betacoronavirus*) adj3 ("19" or "2019")).tw.
21 (covid or covid19).tw.
22 sars cov 2.tw.
23 (ncov or n cov).tw.
24 (novel coronavirus* or novel corona virus*).tw.
25 (CoV 2 or CoV2 or sarscov2 or 2019nCoV or novel CoV or wuhan virus).tw.
26 (coronavirus infection/ or severe acute respiratory syndrome/) and (pandemic/ or pandemic*.tw.)
27 limit 26 to yr="2019 -Current"
28 or/18-25,27
29 infant mortality/
30 newborn mortality/
31 perinatal mortality/
32 perinatal death/
33 ((infant* or baby or babies or neonat* or newborn*) adj2 (death* or mortalit*)).tw.

For peer review only

34 or/29-33
35 28 and 34
36 35 use emczd Embase
37 17 or 36
38 remove duplicates from 37
39 38 use medall
40 38 use emczd

Web of Science – June 23, 2021

5 #4 AND #3
Indexes=SCI-EXPANDED, CPCI-S, ESCI Timespan=1900-2021

4 **TOPIC:** ((infant* or baby or babies or neonat* or newborn*) NEAR/5 (death* or mortalit*))
Indexes=SCI-EXPANDED, CPCI-S, ESCI Timespan=1900-2021

3 #2 OR #1
Indexes=SCI-EXPANDED, CPCI-S, ESCI Timespan=1900-2021

2 **TOPIC:** (sars cov 2 OR sars cov2) *OR* **TOPIC:** (ncov or n cov) *OR* **TOPIC:** (novel coronavirus or novel corona virus)
Indexes=SCI-EXPANDED, CPCI-S, ESCI Timespan=1900-2021

1 **TOPIC:** (covid OR covid19 or covid2019)
Indexes=SCI-EXPANDED, CPCI-S, ESCI Timespan=1900-2021

Cinahl – June 23, 2021

#	Query
S1	(MH "COVID-19")
S2	(MH "COVID-19 Pandemic")
S3	(MH "SARS-CoV-2")
S4	TI covid* OR AB covid* OR TI sars cov 2 OR AB sars cov 2 OR TI novel coronavirus OR AB novel coronavirus OR TI wuhan virus OR AB wuhan virus OR TI 2019nCoV OR AB 2019nCoV
S5	TI (CoV 2 or CoV2 or sarscov2) OR AB (CoV 2 or CoV2 or sarscov2)
S6	S1 OR S2 OR S3 OR S4 OR S5
S7	(MH "Infant Mortality")

For peer review only

S8	(MH "Infant Death")
S9	(MH "Perinatal Death")
S10	TI (((infant* or baby or babies or neonat* or newborn* or perinat*) N3 (death* or mortalit*))) OR AB (((infant* or baby or babies or neonat* or newborn* or perinat*) N3 (death* or mortalit*)))
S11	(S7 OR S8 OR S9 OR S10)
S12	S6 AND S11

For peer review only

Appendix 2: Data Charting Form

Data charting framework

Main category	Subcategory	Description
**Titles**		
**Authors**		
**Year of publication**		
**Study Journal**		
**Study Citation Details**		
**Study objective(s)**		Describe the stated objectives of the studies included in the review
**Study Design**		Specify the types of studies included in the review
**Study Setting**		Specify the geographical areas covered by the studies included in the review<br>Specify the level of medical facility in each study included in the review
**Study Population/Sample size**		Describe the study population and specify the number of participants included in each study
**Eligibility Criteria**		Specify the inclusion and exclusion criteria of each study included in the review
**Study period**		Specify the time period of data collection of each studies included in the review<br>Specify the stage/wave of the pandemic during which each included study was conducted
**Reported primary outcomes (neonates <28 days and infants <1 year-old)**	Frequency of ED visits	Specify the frequency of ED visits before and during the COVID-19 pandemic
	Main Reason(s) for ED visits	Describe the main reasons for ED visits before and during the COVID-19 pandemic
**Reported secondary outcomes (neonates <28 days and infants <1 year-old)**	Change in mortality	Specify if there was an increase or decrease of neonatal/infant mortality during the COVID-19 pandemic compared to pre-pandemic
	Causes of mortality	Describe the main causes of mortality before and during the COVID-19 pandemic
	ED visit length	Specify the ED visit length (in hours) during the COVID-19 pandemic compared to pre-pandemic
	NICU/PICU/HDU admission	Specify the NICU/PICU/HDU admission rate before and during the COVID-19 pandemic
	Reasons for NICU/PICU/HDU admission	Describe the main reasons for NICU/PICU/HDU admission before and during the COVID-19 pandemic

	NICU/PICU/HDU length of stay	Specify the NICU/PICU/HDU length of stay (in days) during the COVID-19 pandemic compared to pre-pandemic
	Infant hospitalization	Specify the infant hospitalization rate before and during the COVID-19 pandemic
	Reasons for infant hospitalization	Describe the main reasons of infant hospitalization before and during the COVID-19 pandemic

For peer review only

Preferred Reporting Items for Systematic reviews and Meta-Analyses extension for Scoping Reviews (PRISMA-ScR) Checklist

SECTION	ITEM	PRISMA-ScR CHECKLIST ITEM	REPORTED ON PAGE #
**TITLE**			
Title	1	Identify the report as a scoping review.	1
**ABSTRACT**			
Structured summary	2	Provide a structured summary that includes (as applicable): background, objectives, eligibility criteria, sources of evidence, charting methods, results, and conclusions that relate to the review questions and objectives.	2
**INTRODUCTION**			
Rationale	3	Describe the rationale for the review in the context of what is already known. Explain why the review questions/objectives lend themselves to a scoping review approach.	3
Objectives	4	Provide an explicit statement of the questions and objectives being addressed with reference to their key elements (e.g., population or participants, concepts, and context) or other relevant key elements used to conceptualize the review questions and/or objectives.	3
**METHODS**			
Protocol and registration	5	Indicate whether a review protocol exists; state if and where it can be accessed (e.g., a Web address); and if available, provide registration information, including the registration number.	n/a
Eligibility criteria	6	Specify characteristics of the sources of evidence used as eligibility criteria (e.g., years considered, language, and publication status), and provide a rationale.	4, 5
Information sources*	7	Describe all information sources in the search (e.g., databases with dates of coverage and contact with authors to identify additional sources), as well as the date the most recent search was executed.	4
Search	8	Present the full electronic search strategy for at least 1 database, including any limits used, such that it could be repeated.	Supplementary appendix 1
Selection of sources of evidence†	9	State the process for selecting sources of evidence (i.e., screening and eligibility) included in the scoping review.	4,5
Data charting process‡	10	Describe the methods of charting data from the included sources of evidence (e.g., calibrated forms or forms that have been tested by the team before their use, and whether data charting was done independently or in duplicate) and any processes for obtaining and confirming data from investigators.	5, supplementary appendix 2
Data items	11	List and define all variables for which data were sought and any assumptions and simplifications made.	5
Critical appraisal of individual	12	If done, provide a rationale for conducting a critical appraisal of included sources of evidence; describe	n/a

For peer review only - http://bmjopen.bmj.com/site/about/guidelines.xhtml

SECTION	ITEM	PRISMA-ScR CHECKLIST ITEM	REPORTED ON PAGE #
sources of evidence§		the methods used and how this information was used in any data synthesis (if appropriate).	
Synthesis of results	13	Describe the methods of handling and summarizing the data that were charted.	5
**RESULTS**			
Selection of sources of evidence	14	Give numbers of sources of evidence screened, assessed for eligibility, and included in the review, with reasons for exclusions at each stage, ideally using a flow diagram.	n/a
Characteristics of sources of evidence	15	For each source of evidence, present characteristics for which data were charted and provide the citations.	n/a
Critical appraisal within sources of evidence	16	If done, present data on critical appraisal of included sources of evidence (see item 12).	n/a
Results of individual sources of evidence	17	For each included source of evidence, present the relevant data that were charted that relate to the review questions and objectives.	n/a
Synthesis of results	18	Summarize and/or present the charting results as they relate to the review questions and objectives.	n/a
**DISCUSSION**			
Summary of evidence	19	Summarize the main results (including an overview of concepts, themes, and types of evidence available), link to the review questions and objectives, and consider the relevance to key groups.	n/a
Limitations	20	Discuss the limitations of the scoping review process.	2
Conclusions	21	Provide a general interpretation of the results with respect to the review questions and objectives, as well as potential implications and/or next steps.	n/a
**FUNDING**			
Funding	22	Describe sources of funding for the included sources of evidence, as well as sources of funding for the scoping review. Describe the role of the funders of the scoping review.	6

JBI = Joanna Briggs Institute; PRISMA-ScR = Preferred Reporting Items for Systematic reviews and Meta-Analyses extension for Scoping Reviews.
* Where *sources of evidence* (see second footnote) are compiled from, such as bibliographic databases, social media platforms, and Web sites.
† A more inclusive/heterogeneous term used to account for the different types of evidence or data sources (e.g., quantitative and/or qualitative research, expert opinion, and policy documents) that may be eligible in a scoping review as opposed to only studies. This is not to be confused with *information sources* (see first footnote).
‡ The frameworks by Arksey and O'Malley (6) and Levac and colleagues (7) and the JBI guidance (4, 5) refer to the process of data extraction in a scoping review as data charting.
§ The process of systematically examining research evidence to assess its validity, results, and relevance before using it to inform a decision. This term is used for items 12 and 19 instead of "risk of bias" (which is more applicable to systematic reviews of interventions) to include and acknowledge the various sources of evidence that may be used in a scoping review (e.g., quantitative and/or qualitative research, expert opinion, and policy document).

From: Tricco AC, Lillie E, Zarin W, O'Brien KK, Colquhoun H, Levac D, et al. PRISMA Extension for Scoping Reviews (PRISMAScR): Checklist and Explanation. Ann Intern Med. 2018;169:467–473. doi: 10.7326/M18-0850.

For peer review only
